# The Story of the Tides

**Published:** 1882-04

**Authors:** 


### THE STORY OF THE TIDES.

### WHAT THEY TELL OF THE GROWING LENGTH OF THE DAY AND OF THE BIRTH OF THE MOON.

From a scientific point of view the work done by the tides is of unspeakable importance. Whence is this energy derived with which the tides do their work? If the tides are caused by the moon, the energy they possess must also be derived from the moon. This looks plain enough, but unfortunately it is not true. Would it be true to assert that the finger of the rfleman which pulls the trigger supplies the energy with which the rifle bullet is animated? Of course it would not. The energy is derived from the explosion of the gunpowder, and the pulling of the trigger is merely the means by which that energy is liberated. In a somewhat simil[illegible]r manner the tidal wave produced by the moon is the means whereby a part of the energy stored in the earth is compelled to expend itself in work. Let me illustrate this by a comparison between the earth rotating on its axis and the flywheel of an engine. The fly-wheel is a sort of reservoir into which the engine pours it power at each stroke of the piston. The various machines in the mill merely draw off the power from the store accumulated in the fly-wheel. The earth is like a gigantic fly-wheel detached from the engine, though still connected with the machines in the mill. In that mighty fly-wheel a stupendous quantity of energy is stored up, and a stupendous quantity of energy would be given out before that fly-wheel would come to rest. The earth's rotation is the reservoir from whence the tides draw the energy they require for doing work. Hence it is that though the tides are caused by the moon, yet whenever they require energy they draw on the supply ready to hand in the rotation of the earth. The earth differs from the fly-wheel of the engine in a very important point. As the energy is withdrawn from the fly-wheel by the machines in the mill, so it is restored thereto by the power of the steam-engine, and the fly runs uniformly. But the earth is merely the fly-wheel without the engine. When the work done by the tides withdraws energy from the earth, that energy is never restored. It therefore follows that the earth's rotation must be decreasing. This leads to a consequence of the most wonderful importance. It tells us that the speed with which the earth rotates on its axis is diminishing. We can state the result in a manner which has the merits of simplicity and brevity. The tides are increasing the length of the day. At present no doubt the effect of the tides in changing the length of the day is very small. A day now is not appreciably longer than a day a hundred years ago. Even in a thousand years the change in the length of the day is only a fraction of a second. But the importance arises from the fact that the change, slow though it is, lies always in one direction. The day is continually increasing. In millions of years the accumulated effect becomes not only appreciable but even of startling magnitude.

The change in the length of the day must involve a corresponding change in the motion of the moon If the moon acts on the earth and retards the rotation of the earth, so, conversely, does the earth react upon the moon. The earth is tormented by the moon, so it strives to drive away its persecutor. At present the moon revolves round the earth at a distance of about 240,000 miles. The reaction of the earth tends to increase that distance, and to force the moon to revolve in an orbit which is continually getting larger and larger. As thousands of years roll on, the length of the day increases second by second, and the distance of the moon increases mile by mile. A million years ago the day probably contained some minutes less than our present day of twenty-four hours. Our retrospect does not halt here; we at once project our view back to an incredibly remote epoch which was a crisis in the history of our system. It must have been at least 50,000,000 years ago. It may have been very much earlier. This crisis was the interesting occasion when the moon was born. The length of the day was only a very few hours. If we call it three hours we shall not be far from the truth. Perhaps you may think that if we looked back to a still earlier epoch, the day would become still less and finally disappear altogether! This is however not the case. The day can never have been much less than three hours in the present order of things. Everybody knows that the earth is not a sphere, but that there is a protuberance at the equator, so that, as our school books tell us, the earth is shaped like an orange. It is well known that this protuberance is due to the rotation of the earth on its axis, by which the equatorial parts bulge out by centrifugal force. The quicker the earth rotates, the greater is the protuberance. If, however, the rate of rotation exceeds a certain limit the equatorial portions of the earth could no longer cling together. The attraction which unites them would be overcome by centrifugal force, and a general break-up would occur. It can be shown that the rotation of the earth when on the point of rupture corresponds to a length of the day somewhere about the critical value of three hours, which we have already adopted. It is therefore impossible for us to suppose a day much shorter than three hours.

Let us leave the earth for a few minutes, and examine the past history of the moon. We have seen the moon revolves around the earth in an ever-widening orbit, and consequently the moon must in ancient times have been nearer the earth than it is now. No doubt the change is slow. There is not much difference between the orbit of the moon a thousand years ago and the orbit in which the moon is now moving. But when we rise to millions of years the difference becomes very appreciable. Thirty or forty millions of years ago the moon was much closer to the earth than it is at present; very possibly the moon was then only half its present distance. We must, however, look still earlier, to a certain epoch not less than fifty millions of years ago. At that epoch the moon must have been so close to the earth that the two bodies were almost touching. Everybody knows that the moon revolves now around the earth in a period of twenty-seven days. The period depends upon the distance between the earth and the moon. In earlier times the month must have been shorter than our present month. Some millions of years ago the moon completed its journey in a week instead of taking twenty-eight days as at present. Looking back earlier still, we find the month has dwindled down to a day, then down to a few hours, until at that wondrous epoch when the moon was almost touching the earth, the moon spun round the earth once every three hours.

In those ancient times I see our earth to be a noble globe, as it is at present. Yet it is not partly covered with oceans and partly clothed with verdure. The primeval earth seems rather a fiery and half molten mass, where no organic life can dwell. Instead of the atmosphere which we now have, I see a dense mass of vapors in which perhaps all the oceans of the earth are suspended as clouds. I see that the sun still rises and sets to give the succession of day and of night, but the day and the night together only amount to three hours instead of twenty-four. Almost touching the chaotic mass of the earth is another much smaller and equally chaotic body. Around the earth I see this small body rapidly rotating. The two revolve together as if they were bound by invisible bands. The smaller body is the moon.—*Nature.*

